# Flecainide in Ventricular Arrhythmias: From Old Myths to New Perspectives

**DOI:** 10.3390/jcm10163696

**Published:** 2021-08-20

**Authors:** Carlo Lavalle, Sara Trivigno, Giampaolo Vetta, Michele Magnocavallo, Marco Valerio Mariani, Luca Santini, Giovanni Battista Forleo, Massimo Grimaldi, Roberto Badagliacca, Luigi Lanata, Renato Pietro Ricci

**Affiliations:** 1 Department of Cardiovascular, Respiratory, Nephrological, Anesthesiological and Geriatric Sciences, Sapienza University of Rome, Policlinico Umberto I, 00161 Rome, Italy; Sara.trivigno3@gmail.com (S.T.); giampaolo.vetta7@gmail.com (G.V.); michelefg91@gmail.com (M.M.); marcoval.mariani@gmail.com (M.V.M.); roberto.badagliacca@uniroma1.it (R.B.); 2 Department of Cardiology, Ospedale GB Grassi, 00121 Ostia, Italy; lucasantinimd@gmail.com; 3 Department of Cardiology, Azienda Ospedaliera-Universitaria “Luigi Sacco”, 20057 Milan, Italy; forleo@me.com; 4 Department of Cardiology, Ospedale Generale Regionale F. Miulli, Acquaviva delle Fonti, 70021 Bari, Italy; fiatric@hotmail.com; 5 Medical Affairs Department, Dompé Farmaceutici SpA, 20057 Milan, Italy; Luigi.Lanata@dompe.com; 6 Centro Cardio-Aritmologico, 00152 Rome, Italy; renatopietroricci@gmail.com

**Keywords:** flecainide, flecainide controlled-release, class IC antiarrhythmic drug, CAST, ventricular arrhythmias, premature ventricular contraction, ventricular tachycardia

## Abstract

Flecainide is an IC antiarrhythmic drug (AAD) that received in 1984 Food and Drug Administration approval for the treatment of sustained ventricular tachycardia (VT) and subsequently for rhythm control of atrial fibrillation (AF). Currently, flecainide is mainly employed for sinus rhythm maintenance in AF and the treatment of idiopathic ventricular arrhythmias (IVA) in absence of ischaemic and structural heart disease on the basis of CAST data. Recent studies enrolling patients with different structural heart diseases demonstrated good effectiveness and safety profile of flecainide. The purpose of this review is to assess current evidence for appropriate and safe use of flecainide, 30 years after CAST data, in the light of new diagnostic and therapeutic tools in the field of ischaemic and non-ischaemic heart disease.

## 1. Introduction

Flecainide is a class IC antiarrhythmic drug (AAD) that was first synthesized in 1972. It was approved by Food and Drug Administration in 1984 for the treatment of symptomatic sustained Ventricular Tachycardia (VT), with a 90% efficacy and without significant adverse events [1,2]. Nowadays flecainide is mainly used for pharmacological conversion and sinus rhythm maintenance in patients with atrial fibrillation (AF) or supraventricular tachycardias [3,4]. The Cardiac Arrhythmia Suppression Trial (CAST) investigated the impact of class IC antiarrhythmic treatment on morbidity and mortality in patients with reduced ejection fraction and frequent premature ventricular complexes (PVC) after myocardial infarction (MI). Published in its preliminary form in 1989, CAST study recorded significantly higher mortality among patients treated with IC AADs compared to placebo and was prematurely dismissed [5].

The CAST study provided a major revision of the role of flecainide, which is therefore recommended in selected patients with preserved systolic function and without ischaemic heart disease [6]. Otherwise, flecainide is contraindicated in patients with previous MI even with preserved ejection fraction and should not be used in case of proved inducible ischaemia. In addition, the current guidelines extended the CAST findings to non-ischaemic structural heart disease despite limited evidence [6].

Thus, the CAST significantly affected and restricted the current use of flecainide in clinical practice causing a fall in employment of class IC AADs in favor of class III AADs [7,8] and exposing patients to the numerous adverse reactions and toxicities of these drugs, in the absence of a robust evidence [9,10].

It is important to underline the paucity of available regarding patients with subcritical coronary artery disease (CAD) without previous MI or inducible ischemia with preserved systolic function and in patients with non-ischaemic structural heart disease. On the other side, recent studies demonstrated safety and efficacy of flecainide in patients with extrasystole-induced cardiomyopathy or Arrhythmogenic Right Ventricular Cardiomyopathy (ARVC) and no Left Ventricular (LV) dysfunction [11,12,13,14]. This review aims to do a critical appraisal of the use of flecainide in Ventricular Arrhythmia management in the light of the current evidence: is it still right to exclude flecainide from treatment for all patients with structural heart disease after 30 years?

## 2. Epidemiology of Ventricular Arrhythmia

VT is commonly seen in medical practice, not rarely in its most feared consequence, Sudden Cardiac Death (SCD). The American Heart Association (AHA) reports over 550,000 annual cardiac arrests, representing half of all cardiovascular deaths [15].

Most patients with sustained VT have underlying structural heart disease such as prior MI, dilated non-ischaemic cardiomyopathy, cardiac sarcoidosis and ARVC [15]. The overall incidence of sustained VT during an acute MI is approximately 10.2% with a high in hospital mortality rate up to 27% [16]. VT in patients with structural heart disease often causes deleterious haemodynamic effects and is correlated with an increased risk of SCD, particularly in elderly [17].

VT can also arise in otherwise normal hearts, Idiopathic Ventricular Arrhythmias (IVA); idiopathic PVCs are the most common arrhythmia in patients with normal heart: approximately 40% of adults experience PVCs on 24-h Holter monitoring [18]. IVAs may be classified in several subtypes according to the origin site. The ventricular outflow tracts are the most common origins of IVAs. Approximately 50% of IVAs originate from the Right Ventricular Outflow Tract (RVOT) [19]. Other origins include the Left Ventricular Outflow Tract (LVOT) (15%), the epicardial myocardium, the great cardiac veins and rarely the pulmonary artery [6,19].

Idiopathic left VTs include verapamil-sensitive left fascicular VT (10%), Bundle Branch Reentry Tachycardia (BBRT) (6%) and rarely interfascicular VT and focal Purkinje VT [6,20].

In a minority of patients VTs or PVCs may arise from the right or left ventricular papillary muscles (7%) and from the mitral or tricuspid annulus (10%) [6,21].

## 3. Mechanisms and Clinical Evaluation of Ventricular Arrhythmia

### 3.1. Pathogenesis

Overall, three mechanisms could be the cause of ventricular arrhythmias: abnormal automaticity, triggered activity and reentry [22,23].

Abnormal automaticity refers to spontaneous depolarization of cells without pacemaker function, as working atrial and ventricular myocardial cells, due to a decrease of membrane potential. Abnormal automaticity is the leading cause of accelerated idioventricular rhythm and it’s caused by ischaemia, electrolyte disturbances and drugs increasing adrenergic tone [24].

The triggered activity results from early or delayed afterdepolarization in ventricular myocytes during phases 2 and 3 or phase 4 of the action potential. It occurs as a result of cyclic adenosine monophosphate dependent diastolic release of intracellular calcium; idiopathic RVOT tachycardia is an example of triggered activity [25,26].

Reentry is the most frequent mechanism causing serious ventricular arrhythmias and occurs when a PVC encounters a heterogeneously conductive substrate within the ventricular myocardium, consisting of two conduction pathways with a different conduction velocity and refractory period. Reentry is the postulated mechanism for VT occurring in a patient with an established scar from prior infarction [27]. VT in non-ischaemic cardiomyopathy is usually due to reentry and is mediated by idiopathic patchy intramural scar. In the case of hypertrophic cardiomyopathy, VT often originates from reentry circuits at the septal level or in the area of localization of hypertrophy at the level of the LV [28].

### 3.2. Clinical Presentation and Electrocardiographic Morphology

During IVAs haemodynamic impairment or serious symptoms are unusual, but patients can have palpitations, atypical chest pain or LV dysfunction. IVAs are distinguished by whether or not they originate from the outflow tracts and display specific electrocardiographic features [29].

**Figure 1:**
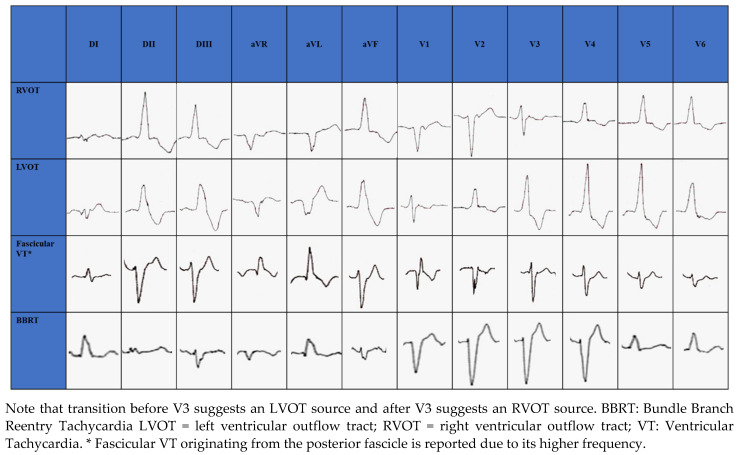
Electrocardiographic morphology of RVOT, LVOT, Fascicular VT and BBRT.

The RVOT is the most common site of origin of idiopathic outflow tract ventricular arrhythmias and is characterized on electrocardiogram (ECG) by a positive QRS complex in the inferior leads and a Left Bundle Branch Block (LBBB) morphology. The origins of RVOT and LVOT can be distinguished on the ECG by the precordial R-wave transition from a predominate S wave to a predominate R wave (rS to Rs). Transitions before V3 indicate an LVOT source and after V3 indicate an RVOT source [29,30,31,32], as seen in Figure 1. When LBBB QRS morphology is observed and the precordial transition occurs at V3, comparison to the patient’s sinus rhythm precordial transition can be useful: a V2 transition ratio ≥ 0.60 suggests LVOT origin with high sensitivity and specificity [33,34].

Among non-outflow tract IVAs, LV papillary muscles are a common site of origin. The postero-medial papillary muscle origin, which is the most common, is characterized by RBBB morphology with superior axis, while the antero-lateral papillary muscle origin is characterized by RBBB and inferior axis [35].

In structurally normal hearts, fascicular VT is generally caused by reentry circuit [36]; ECG morphology changes according to the exit site of the circuit from the retrograde-conducting fascicle: RBBB appearance with a left axis deviation if fascicular VT exits from the left posterior fascicle (90%) (Figure 1); RBBB appearance with a right axis deviation if VT exits from the left anterior fascicle [36]. Simultaneous anterograde activation of the left anterior and posterior fascicles with slow retrograde conduction through a separate septal fascicle can lead to upper septal fascicular VT (<1%), with narrow QRS (QRS < 110 ms) and normal axis [36,37].

BBRT is a macro reentrant tachycardia which uses the bundle branches as reentrant circuit pathways and is typically described in patients with bundle branch block. The most common morphology is the LBBB with a QRS ≥ 160 ms [6] (Figure 1).

## 4. Flecainide Pharmacology

### 4.1. Pharmacodynamics

**Figure 2:**
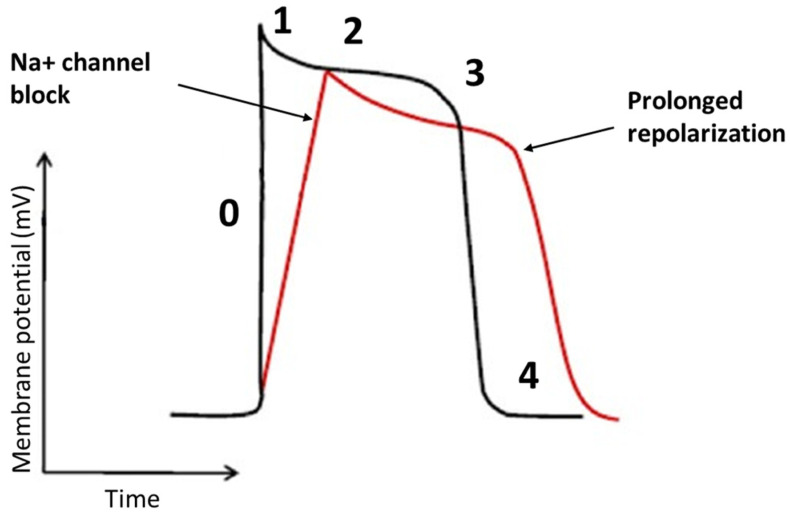
The effects of the flecainide on the cardiac action potential. The main effect of flecainide is a reduction in the slope of phase 0 with a minimal elongation of action potential.

Flecainide binds to open-state of fast inward Na^+^ channel Nav 1.5 in a rate and voltage-dependent manner [38]. The main result of flecainide is a reduction in the slope of phase 0 of monophasic action potential of His-Purkinje tissue and ventricular myocardium resulting in a slow-down of the conduction velocity with a prolongation of HV and QRS intervals, as seen in Figure 2. During a resting heart rate and in a healthy myocardium, the prolongation of HV and QRS is about 10%. The flecainide effect on slowing conduction velocity is proportional to heart rate [38]. Moreover, it is also very effective on areas already characterized by very slow conduction velocity. Usually, VTs with a low heart rate are caused by very slow conduction isthmuses and flecainide is very effective in these cases as it slows down conduction until the tachycardia is interrupted.

In addition flecainide has an inhibitory action on the rapid component of the delayed rectifier K^+^ current and K^ito^ channels [39]. Globally, flecainide increases the duration of the action potential and the effective refractory period in the myocardium, despite they are both reduced in the Purkinje system, due to the blockade of Na^+^ channels [40]. On the other hand, flecainide is highly efficient in the treatment of Catecholaminergic Polymorphic Ventricular Tachycardia (CPVT), which is usually characterized by a high heart rate [41]. In this arrhythmia, however, the efficacy seems to be due to a different mechanism of action. Indeed, CPVT is induced by calcium overload due to sympathetic activation and to a diastolic calcium release from the sarcoplasmic reticulum through defective leaking ryanodine receptor (RYR) 2 channels. Flecainide blocks the RYR2 channels allowing direct targeting of the molecular defect [42].

**Figure 3:**
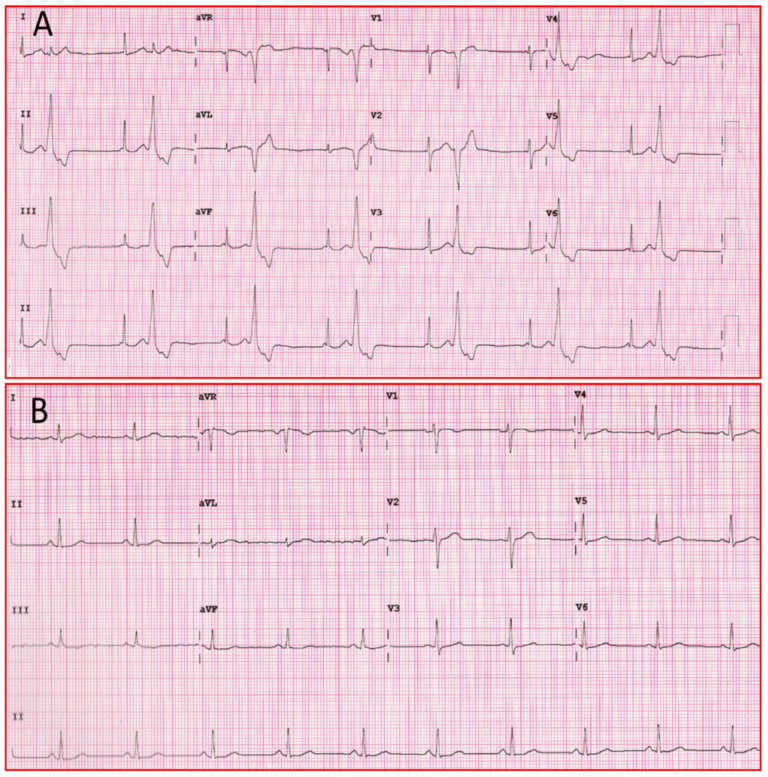
(**A**) Twelve-lead ECG of patient with ventricular bigeminism. (**B**) Twelve-lead ECG of the same patient one hour after taking a flecainide tablet. ECG: electrocardiogram.

Above mechanisms of action contribute to the efficacy of the drug in the treatment of ventricular arrhythmias, as seen in Figure 3.

Flecainide is also effective in Long QT Syndrome (LQT) type 3 as it inhibits not only the peak but also the late component of the Na^+^ current, causing a shortening of the QT interval. This form, indeed, is caused by mutations that increase the late Na^+^ current [43]. In addition, flecainide reduces Na^+^ and Ca^2+^ influx into myocardiocytes causing a negative inotropic effect and a reduction of cardiac output, particularly in patients with CAD or LV dysfunction [38].

Flecainide, due to its pharmacodynamics, widens the PR (17–29%), QT interval (3–8%) and QRS complex (11–27%) [44,45]. In 3–5% of cases flecainide causes conversion of AF to atrial flutter with slow atrial rate leading to 1:1 atrioventricular conduction with high ventricular response [46]. However, this phenomenon can be prevented by concomitant therapy with negative dromotropic agents (beta-blockers, verapamil, diltiazem or digoxin) [47]. The main non-cardiac side effects are dizziness and visual disturbances as a result of action of flecainide on Na channels, while headache and gastrointestinal disturbances are less frequent [48].

### 4.2. Pharmacokinetics

The gastrointestinal tract almost entirely absorbs flecainide, which has bioavailability of 85–90% and achieves peak concentration in approximately 1–3 h [49]. Flecainide has a large volume of distribution with 40% of the drug bound to plasma proteins [49]. Therapeutic plasma levels range between 0.2 and 1.0 mg/mL, higher concentrations are associated with proarrhythmic side effects. Flecainide is metabolized by liver cytochromes CYP2D6 and CYP1A2 and then eliminated in urine [50]. The half-life is in the range of 12–27 h and may last up to 70 h in patients with heart failure, renal disease (creatinine clearance < 50 mL/min) liver disease and advanced age [44]. CYP2D6 genotype is a determinant factor of age-related decline in metabolic clearance of flecainide, resulting in a more prominent effect of the CYP2D6 genotype in elderly [51]. Moreover, the therapeutic range of serum concentration is lower in SCN5A promoter haplotype B carriers than in the wild-type haplotype A homozygotes [52]. Concerning drug interactions, CYP2D6 inducers (carbamazepine, phenytoin, phenobarbital, primidone) increase the elimination rate of flecainide [53,54].On the other hand, CYP2D6 inhibitors as amiodarone, protease inhibitors (amprenavir, darunavir, fosamprenavir, indinavir, lopinavir, ritonavir, saquinavir, tipranavir) [55,56], selective serotonin reuptake inhibitors (citalopram, fluoxetine, paroxetine, sertraline) [57] and serotonin-norepinephrine reuptake inhibitors (duloxetine, venlafaxine) increase flecainide plasma concentration and half-life [58].

### 4.3. Controlled Release Flecainide

Controlled-release flecainide has a reduced and delayed maximum concentration (26 h) and less fluctuations in blood levels than the immediate-release form, allowing once-daily administration. The steady-state blood level is reached after 4–5 days and ranges from 0.27 to 0.33 mcg/mL, far from the blood level at risk of adverse reactions. Thus, the controlled-release form increases treatment compliance and lowers the risk of adverse events and interactions with other drugs while maintaining clinical efficacy [59].

## 5. Guidelines

Current Guidelines for the management of patients with ventricular arrhythmias and the prevention of sudden death have been published in 2015 by the European Society of Cardiology (ESC) [6] and in 2017 by the AHA/American College of Cardiology/Heart Rhythm Society (ACC/HRS) [15,60]. Update of the European recommendations is expected for 2022.

Except for beta-blockers, these documents state that there is no evidence from randomized controlled trials that AADs for ventricular arrhythmias increase survival when used for primary or secondary prevention of SCD [6,15]. Furthermore, because of potential adverse effects of anti-arrhythmic drugs, they must be used with caution. However, AADs are crucial in some patients to control ventricular arrhythmias and improve symptoms.

Flecainide at immediate or controlled release is recommended for treatment of PVC and of VT in absence of structural heart disease and in some specific forms of genetic channelopathies, namely CPVT, LQTS type 3 and Andersen–Tawil Syndrome (LQTS type 7).

### 5.1. Ventricular Tachycardias in Structurally Normal Hearts

In patients with IVAs, PVC/VT treatment is recommended only in symptomatic patients or in case of decline of LV function. Class IC AADs may be an alternative to radiofrequency catheter ablation in such patients when catheter ablation is too complex, not available, not desired or unsuccessful [6]. Catheter ablation should be preferred to antiarrhythmics in patients with RVOT VT, idiopathic left VT and in those refusing long term drug therapy [6]. Detail of recommendations in different clinical setting is reported in Table 1.

### 5.2. CPVT

It is a rare congenital arrhythmogenic disorder triggered by physical or emotional stress. It mainly affects children and younger adults and is characterized by rapid polymorphic and bidirectional VT. Symptoms can range from palpitations and presyncope to syncope and SCD. CPVT is caused by the perturbations in Ca ion handling in the sarcoplasmic reticulum of cardiac myocytes [61]. The two main genotypes are CPVT1 (Mutations in RYR2) and CPVT2 (Mutations in the calsequestrin isoform 2 gene CASQ). Several studies demonstrated that in CPVT-patients flecainide added to beta blockers reduces clinical events, exercise-induced ventricular arrhythmias and defibrillator shocks, with good tolerance, independently from the genotype and even in genotype negative patients [61,62,63,64,65]. Flecainide should be added to betablockers in case of recurrent syncope or bidirectional VT with a class I recommendation in the American guidelines and a class IIA recommendation in the European guidelines (Table 1).

### 5.3. Long QT Syndrome Type 3

Literature showed that some sodium current blockers (mainly class IB mexiletine and class IC flecainide) inhibit the peak sodium current and the late component of the sodium current. Consequently, these drugs can lead to a shortening of the QT interval in patients with LQTS type 3 because this syndrome is caused by mutations that enhance the late sodium current.

Clinical studies in SCN5A mutation carriers demonstrated that flecainide significantly shorten the QTc with no cardiac events during long-term follow up [66,67]. Sodium channel blockers (flecainide, mexiletine, ranolazine) as add-on to shorten QTc > 500 ms have a class IIB recommendation in the European guidelines (Table 1).

### 5.4. Andersen-Tawil Syndrome (ATS)–LQTS 7

Andersen-Tawil syndrome is a rare disorder characterized by a triad of ventricular arrhythmias, including bidirectional and polymorphic VT, dysmorphic features and periodic paralysis. QT prolongation due to abnormal U wave with T+U fusion is the key feature. About 60% are caused by mutations in the KCNJ2 gene. Preliminary studies demonstrated successful use of flecainide in ventricular arrhythmia control in this setting [67].

In summary, immediate-release or controlled-release flecainide plays a key role in management of PVC and VT in structurally normal hearts with a class I indication and should be employed as first line therapy in these patients beside radiofrequency ablation. Choice between drug therapy and ablation is based on patient choice and procedure complexity and risks. Flecainide may be useful as add-on therapy in management of some rare channelopathies such as CPVT and some subtypes of LQTS.

## 6. Flecainide: Evidence Gap and Future Perspective

Current Guidelines for the management of patients with ventricular arrhythmias and the prevention of sudden death state that the choice of a specific drug should be based on the underlying type and severity of concurrent heart disease [6,15]. Specifically, flecainide, as well as other IC AADs, is indicated in the absence of ischaemic heart disease, LV systolic dysfunction or structural heart disease [6,15]. However, is evidence strong enough to forbid this drug in this large subset of patients?

### 6.1. Ischaemic Heart Disease

The prohibition of flecainide in patients with ischaemic heart disease is based on the results of the CAST [5] which demonstrated that flecainide is associated with increased mortality in patients with prior MI and LV dysfunction. This study severely influenced and restricted the current use of flecainide in clinical practice for decades, causing a fall in employment of class IC AADs in favor of class III AADs [7,8] and exposing patients to the numerous adverse reactions and toxicities of these drugs [9]. Specifically, in the CAST trial, 1498 patients with prior MI within 6 days to 2 years before enrollment and with LVEF ≤ 0.55, at least 6 PVCs per hour and no episodes of VT ≥ 15 beats or with rate above 120 bpm were randomized either to flecainide or encainide or placebo treatment. The study was terminated early because of high mortality in patients treated with IC AADs. Myocardial ischemia, as well as congestive heart failure and accumulation of the drug, seem to dynamically promote Na^+^ channel blockade by class IC AADs, thereby favoring a late pro-arrhythmic effect, causing ventricular fibrillation, as well as promoting a negative inotropic effects related to reduced Na^+^ entry (with subsequent reduced Ca^2+^ entry into the myocardial cells) and blocked intracellular interaction between Ca^2+^ and the RYR [53]. However, several criticisms could be made to this study, designed and performed before the thrombolytic and primary percutaneous coronary intervention (PCI) era. Indeed, the study did not include patients with complete revascularization of the culprit lesion that potentially reduces pro-arrhythmic post-MI events and long-term mortality as well as significant ventricular post-MI dysfunction over time. Furthermore, the increased mortality described in the CAST trial was mostly related to flecainide-induced excess of proarrhythmic events in elderly patients with significant pre-existing cardiovascular comorbidity, LV dysfunction and ventricular arrhythmias, rather than having an absolute relation with myocardial ischemia per se [68]. In CAST subanalyses, the presence of non–Q-wave infarction and angina were associated with death, suggesting that a drug–ischaemia interaction rather than a drug–scar interaction mediates the risk for death, supporting a link between ischaemia and electrical instability [69]. In fact, supporting this hypothesis, we have to mention the European Rythmol/Rytmonorm Atrial Fibrillation Trial (ERAFT), which included patients with previous MI but without unstable angina, that did not demonstrate an increase in mortality and ventricular arrhythmias in patients treated with IC AADs [70].

As a result, patients with CAD, preserved ejection fraction, no previous MI and no evidence of inducible ischaemia represents a gray area where the absolute paucity of scientific evidence unjustifiably limits its widespread use. Indeed, there are no scientific data demonstrating an increased proarrhythmic risk of flecainide among patients with CAD in the absence of underlying scar and myocardial ischaemia. Conversely, the study by Pantlin et al. showed the safety of IC AADs in a population of AF patients with occult CAD on coronary flow capacity at positron emission tomography without history of clinically significant CAD [71]. Moreover, Ashraf et colleagues demonstrated in a retrospective study that there was a similar 10-year survival between patients treated with flecainide with no or minimal CAD, nonobstructive CAD and obstructive CAD. Additionally, there was also no increase in mortality among those who had reversible perfusion defects compared with those without, among patients who underwent myocardial perfusion imaging [72].

Thus, there is a compelling need to update the CAST data given the scientific progress in recent years in which an increasingly widespread use of modern therapeutic tools (thrombolysis and, above all, primary PCI) made it possible to select a large population of patients with CAD without myocardial scar and residual ischaemia and for whom there is no scientific evidence against the use of flecainide.

### 6.2. Non-Ischemic Structural Heart Disease

Current guidelines, despite the limited scientific evidence, prudently extended the results of the CAST study to non-ischaemic structural heart disease [6,15]. Indeed, in a recent survey by European Heart Rhythm Association to evaluate clinical practice in the management of ventricular tachycardia in 88 centers from 12 countries, Amiodarone was chosen by 41% of centers as first-line treatment for sustained VT in patients with non-ischaemic structural disease, while only 5.8% of centers chose other AADs [73].

However, several recent studies showed encouraging results regarding the employment of flecainide in patients with non-ischaemic structural heart disease. In this regard, a study by Hyman conducted in patients with extrasystole-induced cardiomyopathy in whom catheter ablation was unsuccessful demonstrated, over a mean follow-up of 4 years, the safety and efficacy of IC AADs to suppress PVCs with recovery of LV ejection fraction in the absence of sustained ventricular arrhythmias [11].

Furthermore, in patients with ARVC with implantable cardioverter defibrillator who were refractory to single-agent antiarrhythmic therapy and/or catheter ablation, Ermakov et al. demonstrated that the addition of flecainide with sotalol or metoprolol was effective in the management of recurrent ventricular arrhythmias over a mean follow-up period of 3 years [12]. A randomized double-blind placebo-controlled crossover pilot study is currently underway to evaluate the efficacy of flecainide in the reduction of ventricular arrhythmias in ARVC patients with implantable cardioverter defibrillators (ClinicalTrials.gov Identifier: NCT03685149).

In addition, Chung, in a study involving patients with the widespread clinical condition of LV hypertrophy (ventricular wall thickness ≥ 1.4 cm), highlighted that treatment with IC AADs did not increase mortality in AF patients in comparison with patients receiving amiodarone [74]. Moreover, even in patients with hypertrophic obstructive cardiomyopathy, flecainide, in a mean follow-up of 9 years, clearly demonstrated its efficacy in the reduction of LV pressure gradient and symptoms and the burden of non-sustained VT compared with disopyramide, with a ratio of 1:11 [75].

Therefore, it anachronistic to accept as dogma the contraindication to flecainide in patients with non-ischaemic structural heart disease, due either to the paucity of data in this regard and to the studies cited above, that showed an increasing evidence of efficacy and safety in the use of flecainide. Instead, it is more appropriate to seek a more precise definition of structural heart disease, using current diagnostic tools such as cardiac magnetic resonance imaging (CMR). In fact, in a recent prospective study involving 946 consecutive patients with ventricular arrhythmias without cardiac ischemia and apparent structural heart disease on transthoracic echocardiography, CMR allowed the diagnosis of structural heart disease in 241 patients (25.5%) [76].

**Figure 4:**
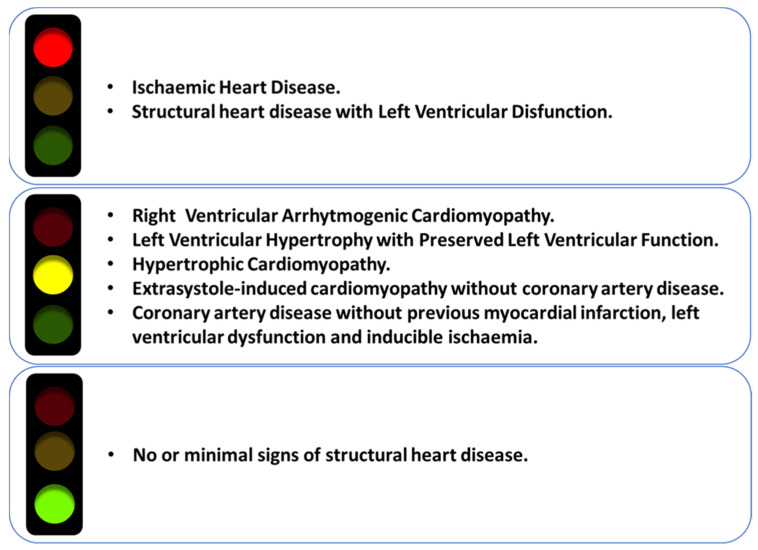
Current evidence on the use of flecainide. Red light: Flecainide is contraindicated. Yellow light: Flecainide may be used in these patients, but further studies are needed. Green light: Flecainide is recommended.

Consequently, the improved assessment of structural heart disease by CMR may allow us to define more precisely the type of structural heart disease that may contraindicate flecainide treatment, reducing the gray area in which the actual paucity of data on the safety of flecainide prevents its possible use but also its contraindication. In the meanwhile, the criteria shown in Figure 4 can help the clinical decision for each patient.

## 7. Conclusions

Flecainide showed its efficacy and safety in the treatment of AF and ventricular arrhythmias. However, flecainide is currently underused because of a restrictive use resulting from an excessively cautious and anachronistic interpretation of the CAST data. In fact, although flecainide is recommended in the treatment of IVAs, it is largely underused in common clinical practice. Conversely, in these patients, the first-line use of amiodarone is often improperly favored, with well-known complications. Even in patients with ischaemic and non-ischaemic structural heart disease, the absolute contraindication to the use of flecainide is widely questionable, also on the basis of modern and widespread diagnostic and therapeutic tools: in fact, in ischaemic heart disease, the spread of primary PCI made it possible to identify a cohort of patients without scar and residual ischaemia who could benefit from the use of flecainide. Future studies will be needed to better define the concept of structural heart disease to avoid a large population of patients who would benefit from the efficacy of flecainide not receiving it.

## Figures and Tables

**Table 1:** Recommendations for immediate-release or controlled-release flecainide use in different clinical setting with the class of recommendation and level of evidence. RVOT = right ventricular outflow tract; LVOT = left ventricular outflow tract; VT = Ventricular Tachycardia; ICD = Implantable Cardioverter Defibrillator; COR = Class of Recommendation; LOE = Level of Evidence; NR = not randomized. * ESC Guidelines; ** AHA Guidelines.

Origin of VT/PVC	Treatment Recommended	COR	LEO
RVOT	Primary Catheter Ablation preferred to Class IC	I	B
LVOT/Aortic Cusp/Epicardial	Class IC first line	I	C
	Catheter Ablation after drug failure	IIA	B
Idiopathic Left VT	Catheter Ablation first line	I	B
	Class IC, beta blockers, verapamil alternative	I	C
Papillary muscle	Class IC, beta blockers, verapamil first line	I	C
	Catheter Ablation after drug failure	IIA	B
Mitral and Tricuspid anulus	Class IC, beta blockers, verapamil first line	I	C
	Catheter Ablation after drug failure	IIA	B
Children with RVOT/LVOT	Class IC alternative to beta blockers/verapamil	IIA	C
	Catheter Ablation after drug failure if >5 years	IIA	B
CPVT	Flecainide should be added to betablockers in case of recurrent syncope or bidirectional VT	IIA * I **	C B-NR
	Flecainide should be added to betablockers to reduce ICD appropriate shocks	IIA	C
LQTS type 3	Sodium channel blockers (flecainide, mexiletine, ranolazine) as add-on to shorten QTc > 500 ms	IIB	C

## Abbreviations

AAD	Antiarrhythmic Drug
ACC	American College of Cardiology
AF	Atrial Fibrillation
AHA	American Heart Association
ARVC	Arrhythmogenic Right Ventricular Cardiomyopathy
BBRT	Bundle Branch Reentry Tachycardia
CAD	Coronary Artery Disease
CMR	Cardiac Magnetic Resonance
CPVT	Catecholaminergic Polymorphic Ventricular Tachycardia
ESC	European Society of Cardiology
ECG	Electrocardiogram
HRS	Heart Rhythm Society
IVA	Idiopathic Ventricular Arrhythmia
LBBB	Left Bundle Branch Block
LQTS	Long QT Syndrome
LV	Left Ventricle
LVOT	Left Ventricular Outflow Tract
MI	Myocardial Infarction
PCI	Percutaneous Coronary Intervention
PVC	Premature Ventricular Complex
RBBB	Right Bundle Branch Block
RVOT	Right Ventricular Outflow Tract
SCD	Sudden Cardiac Death
VT	Ventricular Tachycardia

## References

[1] Hudak J.M., Banitt E.H., Schmid J.R. (1984). Discovery and Development of Flecainide. Am. J. Cardiol..

[2] Hodges M., Haugland J.M., Granrud G., Conard G.J., Asinger R.W., Mikell F.L., Krejci J. (1982). Suppression of Ventricular Ectopic Depolarizations by Flecainide Acetate, a New Antiarrhythmic Agent. Circulation.

[3] Nieuwlaat R., Capucci A., Camm A.J., Olsson S.B., Andresen D., Davies D.W., Cobbe S., Breithardt G., Le Heuzey J.-Y., Prins M.H. (2005). Atrial Fibrillation Management: A Prospective Survey in ESC Member Countries: The Euro Heart Survey on Atrial Fibrillation. Eur. Heart J..

[4] Lavalle C., Magnocavallo M., Straito M., Santini L., Forleo G.B., Grimaldi M., Badagliacca R., Lanata L., Ricci R.P. (2021). Flecainide How and When: A Practical Guide in Supraventricular Arrhythmias. J. Clin. Med..

[5] Echt D.S., Liebson P.R., Mitchell L.B., Peters R.W., Obias-Manno D., Barker A.H., Arensberg D., Baker A., Friedman L., Greene H.L. (1991). Mortality and Morbidity in Patients Receiving Encainide, Flecainide, or Placebo. The Cardiac Arrhythmia Suppression Trial. N. Engl. J. Med..

[6] Priori S.G., Blomström-Lundqvist C., Mazzanti A., Blom N., Borggrefe M., Camm J., Elliott P.M., Fitzsimons D., Hatala R., Hindricks G. (2015). 2015 ESC Guidelines for the Management of Patients with Ventricular Arrhythmias and the Prevention of Sudden Cardiac Death: The Task Force for the Management of Patients with Ventricular Arrhythmias and the Prevention of Sudden Cardiac Death of the European Society of Cardiology (ESC)Endorsed by: Association for European Paediatric and Congenital Cardiology (AEPC). Eur. Heart J..

[7] Anderson J.L., Pratt C.M., Waldo A.L., Karagounis L.A. (1997). Impact of the Food and Drug Administration Approval of Flecainide and Encainide on Coronary Artery Disease Mortality: Putting “Deadly Medicine” to the Test. Am. J. Cardiol..

[8] Al-Khatib S.M., LaPointe N.M.A., Curtis L.H., Kramer J.M., Swann J., Honig P., Califf R.M. (2003). Outpatient Prescribing of Antiarrhythmic Drugs from 1995 to 2000. Am. J. Cardiol..

[9] Ruzieh M., Moroi M.K., Aboujamous N.M., Ghahramani M., Naccarelli G.V., Mandrola J., Foy A.J. (2019). Meta-Analysis Comparing the Relative Risk of Adverse Events for Amiodarone Versus Placebo. Am. J. Cardiol..

[10] Di Biase L., Romero J., Du X., Mohanty S., Trivedi C., Della Rocca D.G., Patel K., Sanchez J., Yang R., Alviz I. (2021). Catheter Ablation of Ventricular Tachycardia in Ischemic Cardiomyopathy: Impact of Concomitant Amiodarone Therapy on Short- and Long-Term Clinical Outcomes. Heart Rhythm.

[11] Hyman M.C., Mustin D., Supple G., Schaller R.D., Santangeli P., Arkles J., Lin D., Muser D., Dixit S., Nazarian S. (2018). Class IC Antiarrhythmic Drugs for Suspected Premature Ventricular Contraction–Induced Cardiomyopathy. Heart Rhythm.

[12] Ermakov S., Gerstenfeld E.P., Svetlichnaya Y., Scheinman M.M. (2017). Use of Flecainide in Combination Antiarrhythmic Therapy in Patients with Arrhythmogenic Right Ventricular Cardiomyopathy. Heart Rhythm.

[13] Ermakov S., Hoffmayer K.S., Gerstenfeld E.P., Scheinman M.M. (2014). Combination Drug Therapy for Patients with Intractable Ventricular Tachycardia Associated with Right Ventricular Cardiomyopathy. Pacing Clin. Electrophysiol..

[14] Della Rocca D.G., Santini L., Forleo G.B., Sanniti A., Del Prete A., Lavalle C., Di Biase L., Natale A., Romeo F. (2015). Novel Perspectives on Arrhythmia-Induced Cardiomyopathy: Pathophysiology, Clinical Manifestations and an Update on Invasive Management Strategies. Cardiol. Rev..

[15] Al-Khatib S.M., Stevenson W.G., Ackerman M.J., Bryant W.J., Callans D.J., Curtis A.B., Deal B.J., Dickfeld T., Field M.E., Fonarow G.C. (2018). 2017 AHA/ACC/HRS Guideline for Management of Patients with Ventricular Arrhythmias and the Prevention of Sudden Cardiac Death: A Report of the American College of Cardiology/American Heart Association Task Force on Clinical Practice Guidelines and the Heart Rhythm Society. Circulation.

[16] Lo R., Chia K.K.M., Hsia H.H. (2017). Ventricular Tachycardia in Ischemic Heart Disease. Card. Electrophysiol. Clin..

[17] Markman T.M., Nazarian S. (2019). Treatment of Ventricular Arrhythmias: What’s New?. Trends Cardiovasc. Med..

[18] Hingorani P., Karnad D.R., Rohekar P., Kerkar V., Lokhandwala Y.Y., Kothari S. (2016). Arrhythmias Seen in Baseline 24-Hour Holter ECG Recordings in Healthy Normal Volunteers During Phase 1 Clinical Trials. J. Clin. Pharm..

[19] Latchamsetty R., Yokokawa M., Morady F., Kim H.M., Mathew S., Tilz R., Kuck K.-H., Nagashima K., Tedrow U., Stevenson W.G. (2015). Multicenter Outcomes for Catheter Ablation of Idiopathic Premature Ventricular Complexes. JACC Clin. Electrophysiol..

[20] Caceres J., Jazayeri M., McKinnie J., Avitall B., Denker S.T., Tchou P., Akhtar M. (1989). Sustained Bundle Branch Reentry as a Mechanism of Clinical Tachycardia. Circulation.

[21] Bhatt A.G., Mittal S. (2018). Ventricular Tachycardia in Structurally Normal Hearts. Encyclopedia of Cardiovascular Research and Medicine.

[22] Wit A.L. (1990). Cellular Electrophysiologic Mechanisms of Cardiac Arrhythmias. Cardiol. Clin..

[23] Cabo C., Wit A.L. (1997). Cellular Electrophysiologic Mechanisms of Cardiac Arrhythmias. Cardiol. Clin..

[24] Flinders D.C., Roberts S.D. (2000). Ventricular Arrhythmias. Prim. Care.

[25] AlMahameed S.T., Ziv O. (2019). Ventricular Arrhythmias. Med. Clin. N. Am..

[26] Lerman B.B., Ip J.E., Shah B.K., Thomas G., Liu C.F., Ciaccio E.J., Wit A.L., Cheung J.W., Markowitz S.M. (2014). Mechanism-Specific Effects of Adenosine on Ventricular Tachycardia. J. Cardiovasc. Electrophysiol..

[27] Tschabrunn C.M., Roujol S., Nezafat R., Faulkner-Jones B., Buxton A.E., Josephson M.E., Anter E. (2016). A Swine Model of Infarct-Related Reentrant Ventricular Tachycardia: Electroanatomic, Magnetic Resonance, and Histopathological Characterization. Heart Rhythm.

[28] Bhaskaran A., De Silva K., Rao K., Campbell T., Trivic I., Bennett R.G., Kizana E., Kumar S. (2020). Ventricular Tachycardia Ablation in Non-Ischemic Cardiomyopathy. Korean Circ. J..

[29] Lavalle C., Mariani M.V., Piro A., Straito M., Severino P., Della Rocca D.G., Forleo G.B., Romero J., Di Biase L., Fedele F. (2020). Electrocardiographic Features, Mapping and Ablation of Idiopathic Outflow Tract Ventricular Arrhythmias. J. Interv. Card. Electrophysiol..

[30] Park K.-M., Kim Y.-H., Marchlinski F.E. (2012). Using the Surface Electrocardiogram to Localize the Origin of Idiopathic Ventricular Tachycardia. Pacing Clin. Electrophysiol..

[31] Della Rocca D.G., Gianni C., Mohanty S., Trivedi C., Di Biase L., Natale A. (2018). Localization of Ventricular Arrhythmias for Catheter Ablation: The Role of Surface Electrocardiogram. Card. Electrophysiol. Clin..

[32] Di Biase L., Romero J., Zado E.S., Diaz J.C., Gianni C., Hranitzki P.M., Sanchez J.E., Mohanty S., Al-Ahmad A., Mohanty P. (2019). Variant of Ventricular Outflow Tract Ventricular Arrhythmias Requiring Ablation from Multiple Sites: Intramural Origin. Heart Rhythm.

[33] Betensky B.P., Park R.E., Marchlinski F.E., Hutchinson M.D., Garcia F.C., Dixit S., Callans D.J., Cooper J.M., Bala R., Lin D. (2011). The V(2) Transition Ratio: A New Electrocardiographic Criterion for Distinguishing Left from Right Ventricular Outflow Tract Tachycardia Origin. J. Am. Coll. Cardiol..

[34] Chen Q., Xu J., Gianni C., Trivedi C., Della Rocca D.G., Bassiouny M., Canpolat U., Tapia A.C., Burkhardt J.D., Sanchez J.E. (2020). Simple Electrocardiographic Criteria for Rapid Identification of Wide QRS Complex Tachycardia: The New Limb Lead Algorithm. Heart Rhythm.

[35] Dukkipati S.R., Koruth J.S., Choudry S., Miller M.A., Whang W., Reddy V.Y. (2017). Catheter Ablation of Ventricular Tachycardia in Structural Heart Disease: Indications, Strategies, and Outcomes-Part II. J. Am. Coll. Cardiol..

[36] Sung R.K., Boyden P.A., Scheinman M. (2017). Cellular Physiology and Clinical Manifestations of Fascicular Arrhythmias in Normal Hearts. JACC Clin. Electrophysiol..

[37] Sung R.K., Kim A.M., Tseng Z.H., Han F., Inada K., Tedrow U.B., Viswanathan M.N., Badhwar N., Varosy P.D., Tanel R. (2013). Diagnosis and Ablation of Multiform Fascicular Tachycardia. J. Cardiovasc. Electrophysiol..

[38] Josephson M.A., Ikeda N., Singh B.N. (1984). Effects of Flecainide on Ventricular Function: Clinical and Experimental Correlations. Am. J. Cardiol..

[39] Anno T., Hondeghem L.M. (1990). Interactions of Flecainide with Guinea Pig Cardiac Sodium Channels. Importance of Activation Unblocking to the Voltage Dependence of Recovery. Circ. Res..

[40] Follmer C.H., Colatsky T.J. (1990). Block of Delayed Rectifier Potassium Current, IK, by Flecainide and E-4031 in Cat Ventricular Myocytes. Circulation.

[41] Padfield G.J., AlAhmari L., Lieve K.V.V., AlAhmari T., Roston T.M., Wilde A.A., Krahn A.D., Sanatani S. (2016). Flecainide Monotherapy Is an Option for Selected Patients with Catecholaminergic Polymorphic Ventricular Tachycardia Intolerant of β-Blockade. Heart Rhythm.

[42] Hilliard F.A., Steele D.S., Laver D., Yang Z., Le Marchand S.J., Chopra N., Piston D.W., Huke S., Knollmann B.C. (2010). Flecainide Inhibits Arrhythmogenic Ca^2+^ Waves by Open State Block of Ryanodine Receptor Ca^2+^ Release Channels and Reduction of Ca^2+^ Spark Mass. J. Mol. Cell. Cardiol..

[43] Belardinelli L., Giles W.R., Rajamani S., Karagueuzian H.S., Shryock J.C. (2015). Cardiac Late Na^+^ Current: Proarrhythmic Effects, Roles in Long QT Syndromes, and Pathological Relationship to CaMKII and Oxidative Stress. Heart Rhythm.

[44] Holmes B., Heel R.C. (1985). A Preliminary Review of Its Pharmacodynamic Properties and Therapeutic efficiancy. Drugs.

[45] Roden D.M., Woosley R.L. (1986). Drug Therapy. Flecainide. N. Engl. J. Med..

[46] Crijns H.J., van Gelder I.C., Lie K.I. (1988). Supraventricular Tachycardia Mimicking Ventricular Tachycardia during Flecainide Treatment. Am. J. Cardiol..

[47] Boriani G., Diemberger I., Biffi M., Martignani C., Branzi A. (2004). Pharmacological Cardioversion of Atrial Fibrillation: Current Management and Treatment Options. Drugs.

[48] Gentzkow G.D., Sullivan J.Y. (1984). Extracardiac Adverse Effects of Flecainide. Am. J. Cardiol..

[49] Conard G.J., Ober R.E. (1984). Metabolism of Flecainide. Am. J. Cardiol..

[50] Tjandra-Maga T., Verbesselt R., Hecken A., Mullie A., Schepper P. (1986). Flecainide: Single and Multiple Oral Dose Kinetics, Absolute Bioavailability and Effect of Food and Antacid in Man. Br. J. Clin. Pharmacol..

[51] Zhou S.-F. (2009). Polymorphism of Human Cytochrome P450 2D6 and Its Clinical Significance: Part I. Clin. Pharmacokinet..

[52] Doki K., Homma M., Kuga K., Aonuma K., Kohda Y. (2013). SCN5A Promoter Haplotype Affects the Therapeutic Range for Serum Flecainide Concentration in Asian Patients. Pharm. Genom..

[53] Aliot E., Capucci A., Crijns H.J., Goette A., Tamargo J. (2011). Twenty-Five Years in the Making: Flecainide Is Safe and Effective for the Management of Atrial Fibrillation. Europace.

[54] Tamargo J., Capucci A., Mabo P. (2012). Safety of Flecainide. Drug Saf..

[55] Shea P., Lal R., Kim S.S., Schechtman K., Ruffy R. (1986). Flecainide and Amiodarone Interaction. J. Am. Coll. Cardiol..

[56] Meda Pharmaceuticals Ltd. (2013). Flecainide Acetate—Summary of Product Characteristics (UK). www.Medicines.Org.Uk/Emc/Medicine/3905.

[57] Lim K.S., Cho J.-Y., Jang I.-J., Kim B.-H., Kim J., Jeon J.-Y., Tae Y.-M., Yi S., Eum S., Shin S.-G. (2008). Pharmacokinetic Interaction of Flecainide and Paroxetine in Relation to the CYP2D6*10 Allele in Healthy Korean Subjects. Br. J. Clin. Pharm..

[58] Nemeroff C.B., DeVane C.L., Pollock B.G. (1996). Newer Antidepressants and the Cytochrome P450 System. Am. J. Psychiatry.

[59] Coumel P., Maison-Blanche P., Tarral E., Périer A., Milliez P., Leenhardt A. (2003). Pharmacodynamic Equivalence of Two Flecainide Acetate Formulations in Patients with Paroxysmal Atrial Fibrillation by QRS Analysis of Ambulatory Electrocardiogram. J. Cardiovasc. Pharmacol..

[60] Tarantino N., Della Rocca D., De Leon De La Cruz N., Manheimer E., Magnocavallo M., Lavalle C., Gianni C., Mohanty S., Trivedi C., Al-Ahmad A. (2021). Catheter Ablation of Life-Threatening Ventricular Arrhythmias in Athletes. Medicina.

[61] Kannankeril P.J., Moore J.P., Cerrone M., Priori S.G., Kertesz N.J., Ro P.S., Batra A.S., Kaufman E.S., Fairbrother D.L., Saarel E.V. (2017). Efficacy of Flecainide in the Treatment of Catecholaminergic Polymorphic Ventricular Tachycardia: A Randomized Clinical Trial. JAMA Cardiol..

[62] Khoury A., Marai I., Suleiman M., Blich M., Lorber A., Gepstein L., Boulos M. (2013). Flecainide Therapy Suppresses Exercise-Induced Ventricular Arrhythmias in Patients with CASQ2-Associated Catecholaminergic Polymorphic Ventricular Tachycardia. Heart Rhythm.

[63] Watanabe H., van der Werf C., Roses-Noguer F., Adler A., Sumitomo N., Veltmann C., Rosso R., Bhuiyan Z.A., Bikker H., Kannankeril P.J. (2013). Effects of Flecainide on Exercise-Induced Ventricular Arrhythmias and Recurrences in Genotype-Negative Patients with Catecholaminergic Polymorphic Ventricular Tachycardia. Heart Rhythm.

[64] Wangüemert Pérez F., Hernández Afonso J.S., Groba Marco M.D.V., Caballero Dorta E., Álvarez Acosta L., Campuzano Larrea O., Pérez G., Brugada Terradellas J., Brugada Terradellas R. (2018). Flecainide Reduces Ventricular Arrhythmias in Patients With Genotype RyR2-Positive Catecholaminergic Polymorphic Ventricular Tachycardia. Rev. Esp. Cardiol..

[65] Hwang H.S., Baldo M.P., Rodriguez J.P., Faggioni M., Knollmann B.C. (2019). Efficacy of Flecainide in Catecholaminergic Polymorphic Ventricular Tachycardia Is Mutation-Independent but Reduced by Calcium Overload. Front. Physiol..

[66] Chorin E., Taub R., Medina A., Flint N., Viskin S., Benhorin J. (2018). Long-Term Flecainide Therapy in Type 3 Long QT Syndrome. EP Eur..

[67] Miyamoto K., Aiba T., Kimura H., Hayashi H., Ohno S., Yasuoka C., Tanioka Y., Tsuchiya T., Yoshida Y., Hayashi H. (2015). Efficacy and Safety of Flecainide for Ventricular Arrhythmias in Patients with Andersen-Tawil Syndrome with KCNJ2 Mutations. Heart Rhythm.

[68] Baranchuk A., Morillo C.A., Thoenes M., Ventura R., Connolly S.J., Natale A., Jalife J. (2008). Current Role of Medical Therapy for Prevention or Termination of Atrial Fibrillation. Atrial Fibrillation.

[69] Akiyama T., Pawitan Y., Greenberg H., Kuo C.-S., Reynolds-Haertle R.A. (1991). The CAST Investigators Increased Risk of Death and Cardiac Arrest from Encainide and Flecainide in Patients after Non-Q-Wave Acute Myocardial Infarction in the Cardiac Arrhythmia Suppression Trial. Am. J. Cardiol..

[70] Meinertz T., Lip G.Y.H., Lombardi F., Sadowski Z.P., Kalsch B., Camez A., Hewkin A., Eberle S. (2002). Efficacy and Safety of Propafenone Sustained Release in the Prophylaxis of Symptomatic Paroxysmal Atrial Fibrillation (The European Rythmol/Rytmonorm Atrial Fibrillation Trial [ERAFT] Study). Am. J. Cardiol..

[71] Pantlin P.G., Bober R.M., Bernard M.L., Khatib S., Polin G.M., Rogers P.A., Morin D.P. (2020). Class 1C Antiarrhythmic Drugs in Atrial Fibrillation and Coronary Artery Disease. J. Cardiovasc. Electrophysiol..

[72] Ashraf H., Ko N.K., Ladia V., Agasthi P., Prendiville T., O’Herlihy F., Pujari S.H., Mulpuru S.K., Scott L., Sorajja D. (2021). Use of Flecainide in Stable Coronary Artery Disease: An Analysis of Its Safety in Both Nonobstructive and Obstructive Coronary Artery Disease. Am. J. Cardiovasc. Drugs.

[73] Tilz R.R., Lenarczyk R., Scherr D., Haugaa K.H., Iliodromitis K., Pürerfellner H., Kiliszek M., Dagres N. (2018). Management of Ventricular Tachycardia in the Ablation Era: Results of the European Heart Rhythm Association Survey. EP Eur..

[74] Chung R., Houghtaling P.L., Tchou M., Niebauer M.J., Lindsay B.D., Tchou P.J., Chung M.K. (2014). Left Ventricular Hypertrophy and Antiarrhythmic Drugs in Atrial Fibrillation: Impact on Mortality: LVH, Antiarrhythmic Drugs, and Mortality. Pacing Clin. Electrophysiol..

[75] Haruki S., Minami Y., Suzuki A., Hagiwara N. (2015). Effects of Flecainide on Left Ventricular Pressure Gradient and Symptoms in Obstructive Hypertrophic Cardiomyopathy: A Comparison of Flecainide and Disopyramide. Heart Vessel..

[76] Andreini D., Dello Russo A., Pontone G., Mushtaq S., Conte E., Perchinunno M., Guglielmo M., Coutinho Santos A., Magatelli M., Baggiano A. (2020). CMR for Identifying the Substrate of Ventricular Arrhythmia in Patients With Normal Echocardiography. JACC Cardiovasc. Imaging.

